# Multi-objective flexible job-shop scheduling problem using modified discrete particle swarm optimization

**DOI:** 10.1186/s40064-016-3054-z

**Published:** 2016-08-30

**Authors:** Song Huang, Na Tian, Yan Wang, Zhicheng Ji

**Affiliations:** 1 School of Internet of Things Engineering, Jiangnan University, 1800 Lihu Avenue, Wuxi, Jiangsu Province 214122 China; 2 Engineering Research Center of Internet of Things Technology Applications, Ministry of Education, Jiangnan University, Wuxi, 214122 China

**Keywords:** Flexible job shop scheduling, Particle swarm optimization, Variable neighborhood search, Non-dominated archive update strategy

## Abstract

Taking resource allocation into account, flexible job shop problem (FJSP) is a class of complex scheduling problem in manufacturing system. In order to utilize the machine resources rationally, multi-objective particle swarm optimization (MOPSO) integrating with variable neighborhood search is introduced to address FJSP efficiently. Firstly, the assignment rules (AL) and dispatching rules (DR) are provided to initialize the population. And then special discrete operators are designed to produce new individuals and earliest completion machine (ECM) is adopted in the disturbance operator to escape the optima. Secondly, personal-best archives (cognitive memories) and global-best archive (social memory), which are updated by the predefined non-dominated archive update strategy, are simultaneously designed to preserve non-dominated individuals and select personal-best positions and the global-best position. Finally, three neighborhoods are provided to search the neighborhoods of global-best archive for enhancing local search ability. The proposed algorithm is evaluated by using Kacem instances and Brdata instances, and a comparison with other approaches shows the effectiveness of the proposed algorithm for FJSP.

## Background

Production planning and scheduling problems arise in many production manufacturing systems. Scheduling problems have been investigated for several decades and various dispatching rules are proposed to optimize scheduling problems. Up to present, many different principles are presented to select and discover effective ways from a number of candidate dispatching rules (Geiger et al. 2006; Tay and Ho 2008; Pickardt et al. 2010; Mouelhi-Chibani and Pierreval 2010; Heger et al. 2016). Geiger et al. (2006) employed a fast learning model with automatically selecting dispatching rules for single machine environments. Tay and Ho (2008) used genetic programming to combine and construct dispatching rules for multi-objective flexible job-shop problems. Pickardt et al. (2010) developed a coupling genetic programming to evolve and produce dispatching rules under varied conditions. Heger et al. (2016) proposed a way to dynamically determine parameter settings of dispatching rules on the previous state. Mouelhi-Chibani and Pierreval (2010) adopted a trained neural networks (NN) to dynamically determine the dispatching rules in real-time flexible manufacturing systems. Some other techniques or approaches, such as shifting bottleneck (Wenqi and Aihua 2004), branch and bound (Della Croce et al. 2002; Artigues and Feillet 2008) were also applied to solve simple scheduling problems in the early researches.

FJSP is more close to the realistic situation and frequently used in flexible manufacturing systems. During the past decades, many researchers have a fast-growing interest on FJSP and amount of written works have been published. However, no satisfactory algorithm presently is available for solving the problem to optimality in expected time. In recent years, most researchers have recognized that probabilistic search method is an attractive alternative to solve this constrained optimization problem. Genetic Algorithm (GA) had emerged as one of the most important method to solve discrete optimization problems and many variants of GA were developed to solve FJSP in the published literatures (Cwiek and Nalepa 2014; Li and Chen 2014; Moghadam et al. 2014; Wang et al. 2014; Zhang et al. 2011). A fast GA with a combination of active schedule constructive crossover (ASCX) and generalized order crossover (GOX) was proposed to solve FJSP by Cwiek and Nalepa (2014). In addition, high-low fit selection scheme was developed to enhance the search ability. Li and Chen (2014) presented an improved GA with two level coding, working sequence coding and machine distribution coding. Crossover and mutation operators were well-designed and neighborhood structure was defined to minimize makespan for FJSP. Aiming at makespan of FJSP, Moghadam et al. (2014) presented GA to create active schedule, which used an Operation order-based Global Selection (OGS) to generate high-quality initial population and introduced crossover operator with precedence preserving order-based crossover (POX) and uniform crossover. Then intelligent mutation operator was introduced to GA. To minimize makespan criterion of FJSP, a hybrid GA with modified coding scheme was presented by Wang et al. (2014). In the hybrid GA, a novel machine assignment strategy was proposed in the initial phase and an improvement strategy was performed when current best solution had not been improved. To minimize the makespan, well-designed representation, global selection and local selection for high-quality initial population, crossover and mutation operators in GA were all developed (Zhang et al. 2011).

Tabu Search (TS) and Particle Swarm Optimization (PSO) had also been investigated to optimize the FJSP (Shao et al. 2013; Jia and Hu 2014; Kamble et al. 2015). Path-relinking TS with neighborhood search and back-jump tracking was presented by Jia and Hu (2014). In details, path-relinking technique to generate improved solutions and dimension-oriented intensification search to find better solutions around extreme solutions were introduced. Kamble et al. (2015) presented a hybrid multi-objective PSO and simulated annealing (SA) algorithm to solve five-objective FJSP. Rescheduling strategy was applied to overcome the machine breakdown and then Pareto front and crowding distance were introduced to handle five-objective problems. Identifying an approximation of the Pareto front of FJSP, Shao et al. (2013) developed a hybrid discrete PSO and SA and a novel displacement strategy was embedded to the proposed algorithm. Also, Pareto ranking and crowding distance method were adopted to deal with multi-objective problems.

Other optimization algorithms, such as firefly algorithm (FA) (Karthikeyan et al. 2014), harmony search algorithm (HS) (Yuan et al. 2013; Gao et al. 2014), biogeography-based optimization (BBO) (Rahmati and Zandieh 2012), differential evolution algorithm (DE) (Balaraju et al. 2014), evolutionary algorithm (EA) (Chiang and Lin 2013) and immune algorithm (IA) (Xue et al. 2014) have been used to solve FJSP in recent years. By defining the presentation of attractiveness, the distance and movement of FA, a hybrid discrete FA incorporating local search with neighborhood structures was presented by Karthikeyan et al. (2014) to minimize the makespan, the critical workload and the total workload. In the Yuan’s work (2013), a discrete hybrid harmony search (HHS) embedding a local search procedure, which provided a neighborhood structure based on common critical operations to enhance the local search, was developed to optimize the makespan. Pareto-based grouping discrete harmony search algorithm (PGDHS) was proposed to optimize the makespan and the mean of earliness and tardiness (Gao et al. 2014). Several new heuristics scheme were firstly designed to the initialization of harmony memory. In addition, multiple strategies and local search were proposed to improve the performance of this algorithm.

This paper proposes a well-designed MOPSO algorithm to optimize three-objective FJSP (the makespan, the total workload and the critical machine workload). Firstly, the AL and DR methods from other works are applied to initialize the population. Then an extended position update formula with two-vector discrete operators is designed and the discrete operator *f*_2_ is applied to share the information of personal-best positions and global-best position. Then disturbance operator *f*_3_ is to explore other space. In details, *f*_2_ is applied to cross the current position with the personal-best position with a probability, or the current position with the global-best position with the other probability. Secondly, personal-best archives and global-best archive updated by predefined non-dominated archive update strategy are developed to obtain high-quality and high-diversity positions, and the personal-best position is selected from the corresponding personal-best archive and the global-best position is selected from the global-best archive. Finally, variable neighborhood search is introduced to exploit the global-best archive.

The organization of the rest is as follows: “Problem formulation**”** section briefly describes the problem formulation of FJSP. In “The MOPSO algorithm” section, a brief introduction of basic PSO is given, and then the details of MOPSO algorithm are presented. The simulation in comparison with other algorithms and parameter analysis are shown in “The simulation experiments” section. Finally, “Conclusions” section concludes this paper.

## Problem formulation

### Flexible job-shop scheduling problem

For FJSP, each operation can be assigned to one machine from a set of available machines and then sequenced under precedence constraint. There are a set of *n* jobs *J* = {*J*_1_, *J*_2_, …, *J*_*k*_, …, *J*_*n*_} to be processed on a set of *m* machines, *M* = {*M*_1_, *M*_2_, …, *M*_*k*_,…, *M*_*m*_}. Each job consists of *n*_*i*_ operations, $O_{i} = \{ O_{i1} \ldots ,O_{i2} , \ldots ,O_{ij} , \ldots O_{{in_{i} }} \}$, where *O*_*ij*_ and *n*_*i*_ respectively denote the *jth* operation of job *i *and the number of operations for job *i*. The machine processing the operation *O*_*ij*_ is denoted as *M*_*k*_ from a given available machines called *M*_*ij*_, where *M*_*ij*_ denote the set of available machines for the operation *O*_*ij*_ and *M*_*ij*_ ⊂ *M*. Partial flexibility and total flexibility are two kinds of FJSP. The former means that the available machines *M*_*ij*_ processing on the operation *O*_*ij*_ are a subset of *M*, and the latter means that the available machines *M*_*ij*_ processing on the operation *O*_*ij*_ includes all machines of *M*. Also, *p*_*ijk*_ denotes the executing time of operation *O*_*ij*_ on the machine *M*_*k*_.

Hypotheses are listed as follows: (1) They are all independent jobs and machines. (2) Setting up times of machines and move times between operations are negligible. (3) A machine can only execute one operation at a given time. (4) For the same job, only one operation is processing at the same time. (5) There are no precedence constraints among different jobs. The task is to determine an assignment and a sequence of operations to minimize several scheduling criteria. In this paper, three objectives of scheduling criteria are as follows:

1. *C*_M_: Makespan or maximal completion time of machines.

$$C_{\text{M}} = \mathop {\hbox{max} }\limits_{1 \le k \le m} \{ C_{k} \} \,$$

2. *W*_T_: Total workload of machines, which is the total working time of all machines.

$$W_{\text{T}} = \sum\limits_{i = 1}^{n} {\sum\limits_{j = 1}^{{n_{i} }} {\sum\limits_{k = 1}^{m} {p_{ijk} x_{ijk} } } }$$

3. *W*_M_: Critical machine workload, which is the biggest workload among the machines.

$$W_{\text{M}} = \mathop {\hbox{max} }\limits_{1 \le k \le m} \sum\limits_{i = 1}^{n} {\sum\limits_{j = 1}^{{n_{i} }} {p_{ijk} x_{ijk} } }.$$

### Encoding and decoding

Encoding a scheduling as a two-vector representation, which includes operation sequence vector and machine assignment vector, is an effective way to represent the decision of FJSP. In details, operation sequence vector is a decision of all operations’ order, and machine assignment vector is a decision of the assigned machines of all operations. Direct and indirect encoding scheme are two types of encoding methods for operation sequence representation. Operation-based representation is an effective indirect encoding method for operation sequence vector, which can absolutely meet the constraints and is able to encode a feasible schedule (Gen et al. 1994). Thus, the operation-based encoding scheme is adopted to represent operation sequence vector. In this encoding scheme, the length of each vector equals to the total number of all operations. The number denotes the corresponding job and the *kth* occurrence of the number refers to the *kth* operation of this job. For the machine assignment vector, the numbers represent the machines assigned to the operations with the ascending job number successively.

**Fig. 1:**
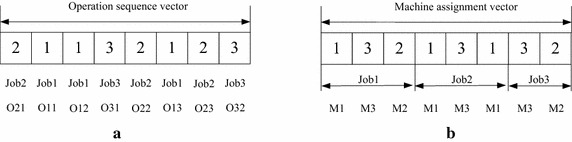
The two-vector representation **a** operation sequence vector, **b** machine assignment vector

Take three-job, three-machine instance for an example. As illustrated in Fig. 1, the operation sequence vector [2 1 1 3 2 1 2 3] represents the operation sequence [*O*_21_, *O*_11_, *O*_12_, *O*_31_, *O*_22_, *O*_13_, *O*_23_, *O*_32_]. Then the machine assignment vector [1 3 2 1 3 1 3 2] represents the operations and their assigned machines: (*O*_11_, *M*_1_), (*O*_12_, *M*_3_), (*O*_13_, *M*_2_), (*O*_21_, *M*_1_), (*O*_22_, *M*_3_), (*O*_23_, *M*_1_), (*O*_31_, *M*_3_), (*O*_32_, *M*_2_). The processing time can be organized in Table 1, where rows correspond to operations and columns correspond to machines. According to Table 1, we can obtain the processing times of the example, which is [5 2 1 1 4 5 3 4].

**Table 1:** Processing times for three-job, three-machine instance

		Processing time		
		*M*_1_	*M*_2_	*M*_3_
*J*_1_	*O*_11_	5	3	–
	*O*_12_	–	1	2
	*O*_13_	3	1	–
*J*_2_	*O*_21_	1	–	4
	*O*_22_	–	5	4
	*O*_23_	5	–	6
*J*_3_	*O*_31_	–	6	3
	*O*_32_	5	4	5

A semi-active schedule often occurs in decoding a schedule and results in the increasing of makespan. An active schedule can avoid the weakness. Local left shift and global left shift are designed to decode a schedule to a active one (Li et al. 2010). In this paper, a left-shift function proposed by Li et al. (2010) is applied to decode a semi-active schedule into an active schedule. After applying the left-shift function to the semi-active schedule, the schedule can be decoded as an active schedule.

## The MOPSO algorithm

In this section, we developed an effective MOPSO algorithm and the details of the proposed algorithm is described as follows: “Initialization” section describes population initialization and “The details of MOPSO algorithm” section presents the extended position update formula in discrete PSO. Then variable neighborhood search is developed in “Variable neighborhood search” section. Furthermore, non-dominated archive update strategy of personal-best archives and global-best archive, and the selection method of the personal-best position and the global-best position are introduced in “Personal-best positions and global-best position” section. Finally, the stop criterion and the flowchart of the proposed algorithm are described in “Stop criterion**”** section.

### Initialization

Appropriate initial methods can provide enough diversity and high-quality individuals to the population. For FJSP, the initialization includes initializing machine assignment and initializing operation sequence. The hybridization of assignment rules (AL) and dispatching rules (DR) from other researchers are proved to be extremely efficient initializing methods for the FJSP (Kacem et al. 2002a; Bagheri et al. 2010; Defersha and Chen 2010; Li et al. 2010). To obtain more promising individuals, the initial population in our study is generated by three AL methods (20 % by GPT, 20 % by LPT, and 60 % by the random rule) proposed by Kacem et al. (2002a) and DR method (Random rule) proposed by Pezzella et al. (2008).

### The details of MOPSO algorithm

#### Discrete PSO

Inspired by fish and birds’ behavior, particle swarm optimizer is developed by Kennedy and Eberhart. As *N* individuals search in *d* dimensions space, individual *i* has a position $\varvec{x}_{i} = (x_{i1} ,x_{i2} , \ldots ,x_{id} )$ and a velocity $\varvec{v}_{i} = (v_{i1} ,v_{i2} , \ldots ,v_{id} )$. The personal-best position of the *ith* individual is $\varvec{p}_{i} = (p_{i1} ,p_{i2} , \ldots ,p_{id} )$ and the global-best position of the population is $\varvec{p}_{\text{g}} = (p_{{{\text{g}}1}} ,p_{{{\text{g}}2}} , \ldots ,p_{{{\text{g}}d}} )$. In the search process, the individual tries to update the velocity and the position using the current velocity, personal-best position and global-best position. Therefore, the velocity $\varvec{v}_{i}$ and the position $\varvec{x}_{i}$ of individual *i* can be manipulated by Eqs. (1) and (2).

1 $$\varvec{v}_{i}^{t + 1} = \omega \varvec{v}_{i}^{t} + c_{1} r_{1} (\varvec{p}_{i}^{t} -\varvec{x}_{i}^{t} ) + c_{2} r_{2} (\varvec{p}_{\text{g}}^{t}-\varvec{x}_{i}^{t})$$

2 $$\varvec{x}_{i}^{t + 1} = \varvec{x}_{i}^{t} + \varvec{v}_{i}^{t + 1}$$

where *c*_1_, *c*_2_ are the coefficient of cognitive and social knowledge. *ω* denotes inertia factor. *r*_1_, *r*_2_ are real numbers in (0, 1). *t* denotes the current generation.

A discrete version of particle swarm optimizer needs to be developed to solve flexible job shop scheduling problem, which is a specific optimization problem with discrete variables. Various discrete operators are developed to deal with discrete variables and it is also a significant problem to obtain appropriate discrete form of particle swarm optimizer. Discrete operators are convenient to handle the discrete variables, and some discrete operators are designed and incorporated into particle swarm optimizer. The position update equation with discrete operators is as follows:

3 $$\varvec{x}_{i}^{t + 1} = \omega \otimes f_{1} (\varvec{x}_{i}^{t} ) + c_{1} \otimes f_{2} (\varvec{x}_{i}^{t} ,\varvec{p}_{i}^{t} ) + c_{2} \otimes f_{2} (\varvec{x}_{i}^{t} ,\varvec{p}_{\text{g}}^{t} )$$

where *ω*, *c*_1_, and *c*_2_ are three probabilities, which represents the impact of current position, personal-best position and global-best position. ⊗ represents the right operator of ⊗ will be implemented while the probability in the left of ⊗ is satisfied and + represents that left term of + is finished and the right term of + starts. Two discrete operator, *f*_1_ and *f*_2_, are well-designed to deal with discrete variables $\varvec{x}_{i}$. In detail, *f*_2_ includes improved precedence operation crossover (IPOX) (Zhang et al. 2005) and multipoint preservative crossover (MPX) (Zhang et al. 2007). Additionally, *c*_2_ equals to $\overline{c}_{1}$, which indicates that the condition *rand* ≤1−*c*_1_ is satisfied. Detail implementation of *f*_1_ and *f*_2_ is given in “The details of *f*_1_, *f*_2_ and *f*_3_” section. *f*_3_ is then embedded to search more space and is also provided in “The details of *f*_1_, *f*_2_ and *f*_3_” section. As above description, the pseudo-code of the Eq. (3) is as follows: 

#### The details of *f*_1_, *f*_2_ and *f*_3_

As the discrete operator *f*_2_ is not applied to all individuals, the function of *f*_1_ is keeping the individuals unchanged with the probability *ω* and otherwise, perturbation operator *f*_3_ is applied to the individuals. Then *f*_2_ is used to obtain useful information from the personal-best positions with the probability *c*_1_ and global-best position with the probability *c*_2_.

*Discrete operator f*_*2*_ The operator *f*_2_ is implemented on the operation sequence vector and the machine assignment vector successively. IPOX is implemented on the operation sequence vector and MPX is implemented on the machine assignment vector.

For example, F_1_ and F_2_ are two parents; S_1_ and S_2_ are their children. The machine assignment vectors remain the same, and the procedure of *f*_2_ (IPOX) on operation sequence vector is as follows:

- Step 1: Select the operation sequence vectors of the parents F_1_ and F_2_, and all the jobs are randomly divided into two set J_1_ and J_2_.
- Step 2: Copy the elements of F_1_ that are included in J_1_ to S_1_ in the same position and copy the elements of F_2_ that are included in J_1_ to S_2_ in the same position.
- Step 3: Copy the elements of F_2_ that are included in J_2_ to S_1_ in the same order and copy the elements of F_1_ that are included in J_2_ to S_2_ in the same order.

The operation sequence vectors remain the same, and the procedure of *f*_2_ on the machine assignment vectors (MPX) is as follows:

- Step 1: Select the machine assignment vectors of the parents F_1_ and F_2_.
- Step 2: Generate a decision vector *H* with random integers 0 and 1, which has the same length with the machine assignment vector.
- Step 3: Find the places which are equal to 1 in *H*, and then copy the machine assignment number in these places of F_1_ and F_2_ to S_2_ and S_1_.
- Step 4: Copy machine numbers of the rest places in F_1_ and F_2_ to S_1_ and S_2_.

**Fig. 2:**
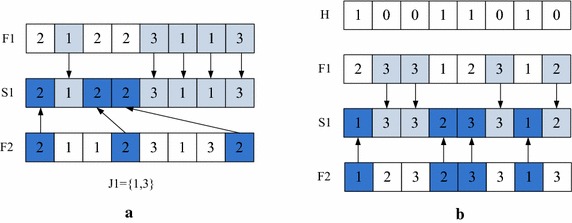
The discrete operator *f*_2_ on operation sequence and machine assignment. **a** IPOX for the operation sequence, **b** MPX for the machine assignment

The IPOX of *f*_2_ works as in Fig. 2(a), and the MPX of *f*_2_ works as in Fig. 2(b).

*Perturbation operator f*_*3*_ In order to avoid premature convergence, the perturbation operator *f*_3_ is adopted. Earliest completion machine (ECM) is an efficient method to assign an operation to a machine (Lin 2015). It can complete the operation with the earliest completion time but it needs expensive time consumption. Consequently, we apply the ECM rule with a small probability *c*_3_. The ECM rule (*f*_3_) works as follows: Calculate complete time of each operation in all machines by the order in operation sequence vector, and then find the machine with shortest time and assign the operation to the machine.

### Variable neighborhood search

Disjunctive graph can also represent a feasible schedule. In the disjunctive graph, the longest path in the disjunctive graph is the critical path and the critical operations are these operations on the critical path. The maximal sequence of joint public critical operations (which are belong to all the critical paths) processed on the same machine is defined as public critical block. Only changing the critical paths can reduce the makespan and neighborhoods based on public critical block theory can significantly reduce the search scope. Therefore, three neighborhood structures based on public critical block are defined in variable neighborhood search. Two neighborhoods of machine moves (NH_1_ and NH_2_) are generated on the critical operation and one neighborhood of operation moves (NH_3_) is generated on public critical block. The details of three neighborhoods are as follows:

*NH*_*1*_ Find these machines *M*_s_ with the maximal makespan and then randomly select a machine *M*_*k*_ from *M*_s_. Randomly select an public critical operation *O*_*ij*_ on the machine *M*_*k*_. From the candidate machine set *M*_*ij*_, randomly select another machine *M*’_*k*_ different from the current one *M*_*k*_ for the selected operation *O*_*ij*_. Then randomly select an insert point, which meets precedence constraints of the same job, from the chosen machine *M*’_*k*_ and insert the operation in this point.

*NH*_*2*_ Randomly select an public critical operation *O*_*ij*_ with more than one candidate machines and sort the candidate machines of *O*_*ij*_ by the processing time in ascending order. Then randomly select another machine *M*’_*k*_, which is different from the current one *M*_*k*_, from the front half candidate machines. Then assign the machine *M*’_*k*_ to the operation *O*_*ij*_.

**Fig. 3:**
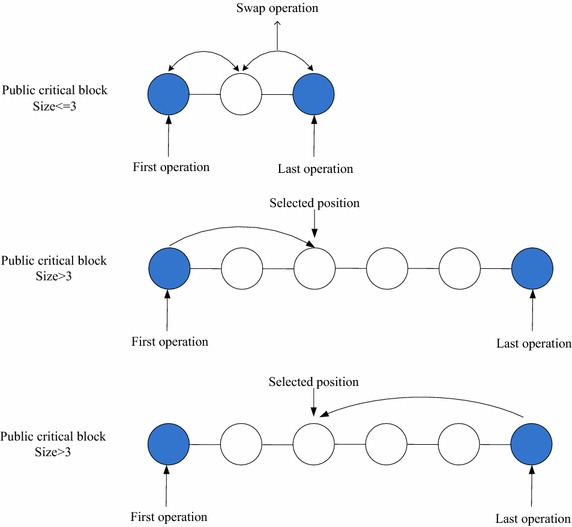
The procedure of NH_3_

*NH*_*3*_ Choose a public critical block *π* randomly, and then randomly select an operation *O*_*i*_^π^ of the block *π*, which is different from the first operation or the last operation of the block *π*. If the size of *π* is equal to 3, swap the first operation or the last operation of the block *π* with the operation *O*_*i*_^π^ as they are not belong to the same job. If the size of *π* is bigger than 3, insert the first operation or the last operation of the block *π* into a random selected position in *π*. The procedure of NH_3_ is illustrated in Fig. 3.

The pseudo-code of variable neighborhood search is given in Algorithm 1. 

where *K* is the number of the neighborhood types and *K* equals to 3. *s*″ ≻ *s* indicates that *s*″ dominates *s*.

The pseudo-code of local search is given in Algorithm 2. where *N*_S_ is the searching size of the neighborhoods and *N*_S_ is set to 20 empirically.

### Personal-best positions and global-best position

#### The personal-best archives and global-best archive

In this section, each individual has a personal-best archive to preserve non-dominated individuals’ positions obtained by its history search and the global-best archive is used to preserve non-dominated individuals’ positions obtained by the population. To obtain high-quality and high-diversity solutions, a selective strategy of non-dominated individuals’ positions should be developed to update the global-best archive and the personal-best archive. Many selection mechanisms, such as NSGA-II (Deb et al. 2002), MOEA/D (Li and Zhang 2009), and SPEA2 (Zitzler et al. 2002) have already been used to sort the non-dominated individuals. Weighted sum approach can combine all the objectives into a single objective to represent relative superiority of individuals, and this method can change the impacts of each criterion via adjusting the weights to solve the multi-objective problems. In our study, a novel weighted sum approach is presented as a criterion to update the personal-best archives and global-best archive.

The update procedure is as follows: For particle *i*, suppose the maximal size of its personal-best archive is *N*_p_. Add the personal-best archive and particle *i* to form a new archive *Ω*, and then select *N*_p_ non-dominated particles from *Ω* by non-dominated archive updating strategy. The global-best archive is updated as follows: Suppose the maximal size of global-best archive is *N*_a_. Add the current global-best archive and the non-dominated particles of the current population to form a new archive *Ω*’, and then select *N*_a_ non-dominated particles from *Ω*’ by non-dominated archive update strategy. Here *N*_p_, *N*_a_ is empirically set to 5 and 15. The non-dominated archive update strategy is implemented as follows:

##### Non-dominated archive update strategy

**Fig. 4:**
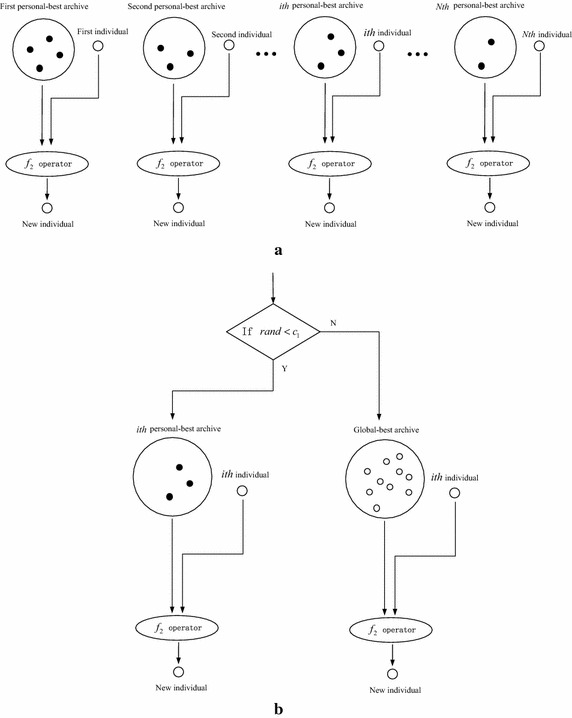
The selection of personal-best position and the global-best position and the function of *f*_2_ operator. **a** *f*_2_ operator on each personal-best archive. **b** Procedure of *f*_2_ operator

Randomly generate three numbers *w*_1_, *w*_2_, *w*_3_ in [0 1] and the weights are able to add some random impacts on the objectives. Then three coefficients (here is the 10, 1, 10^−1^), which represents the real impacts of three objectives, are multiplied by these weights. Suppose the archive is *Ω* and its limited size is *N*_a_. The pseudo-code of the non-dominated archive update strategy integrating weighted sum approach is as follows: 

After personal-best archives and global-best archive are updated by the non-dominated archive update strategy, the personal-best position is randomly selected from its personal-best archive and the global-best position is randomly selected from the global-best archive. The selection strategy of the personal-best position and the global-best position as well as the updating procedure of population is illustrated in Fig. 4.

### Stop criterion

**Fig. 5:**
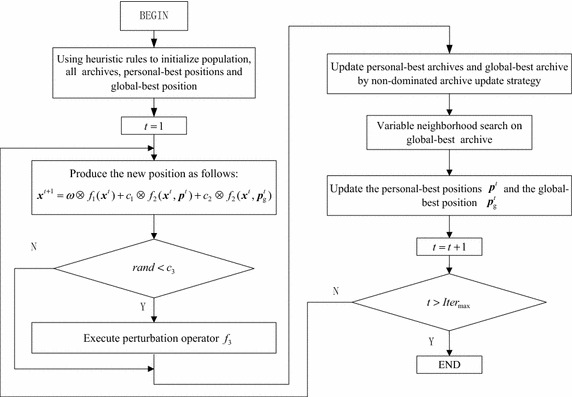
The flowchart of MOPSO

Stop criterion: the predefined number of generations is reached. From the above description, the flowchart of the proposed algorithm is shown in Fig. 5.

## The simulation experiments

### Parameter settings and results

Four Kacem instances and ten Brdata instances (Brandimarte 1993; Kacem et al. 2002b) are used to evaluate the performance of our algorithm and several published algorithms are applied to compared with the proposed algorithm. The proposed algorithm is implemented in Matlab 7.1 on Lenovo PC with 4G RAM and 3.4G Intel (R) Core(TM) i3-3240 CPU. In order to obtain reliable results, our algorithm is run ten times on the same instance. The parameters are chosen experimentally to get a better satisfactory solution. The population size *N* is set as 100. The maximal generation number *Iter*_max_ is set as 300. *ω* is set as 0.98. *c*_1_ and *c*_2_ are set as 0.6, 0.4. *c*_3_ is set as 0.02.

#### Test on the Kacem instances

Firstly, four Kacem instances ranging from 4 jobs × 5 machines to 15 jobs × 10 machines, which are frequently tested on recently published literatures, are used to evaluate the validity and performance. The compared algorithms are the HTSA presented by Li et al. (2010), the AIA presented by Bagheri et al. (2010), the Xing algorithm by Xing et al. (2010), the MOGA by Wang et al. (2010), the P-DABC algorithm presented by Li et al. (2011a), the SEA presented by Chiang and Lin (2013). Table 2 lists non-dominated solutions obtained by the proposed algorithm and several recently published algorithms for four Kacem instances. For the 4 jobs × 5 machines instance, the 8 jobs × 8 machines instance and the 10 jobs × 10 machines instance, all the solutions obtained by seven algorithms are non-dominated solutions. For the 15 jobs × 10 machines instance, the solutions obtained by the HTSA, Xing algorithm and MOPSO algorithm are the same and dominate some solutions obtained by AIA, MOGA and P-DABC.

**Table 2:** Results of the four Kacem instances

Kacem (m × n)	4 × 5			8 × 8			10 × 10			15 × 10		
Objective	*C*_M_	*W*_T_	*W*_M_	*C*_M_	*W*_T_	*W*_M_	*C*_M_	*W*_T_	*W*_M_	*C*_M_	*W*_T_	*W*_M_
HTSA	11	32	10	14	77	12	7	43	5	11	91	11
	12	32	8	15	75	12	7	42	6	11	93	10
AIA		N/A		14	77	12	7	43	5	11	93	11
Xing	12	32	8	14	77	12	7	42	6	11	91	11
		N/A		15	76	12	8	42	5	11	93	10
MOGA	11	32	10	15	81	11	8	42	5	11	91	11
	12	32	8	15	75	12	7	42	6	12	95	10
	11	34	9	16	73	13	8	41	7	11	98	10
P-DABC	11	32	10	14	77	12	8	41	7	12	91	11
	12	32	8	15	75	12	7	43	5	11	93	11
	13	33	7	16	73	13	8	42	5		N/A	
		N/A			N/A			N/A			N/A	
SEA		N/A		14	77	12		N/A			N/A	
		N/A		15	75	12		N/A			N/A	
		N/A		16	73	13		N/A			N/A	
		N/A		16	77	11		N/A			N/A	
MOPSO	11	32	10	16	73	13	8	41	7	11	91	11
	13	33	7	14	77	12	8	42	5	11	93	10
	12	32	8	16	77	11	7	43	5		N/A	
		N/A		15	75	12	7	42	6		N/A	

**Fig. 6:**
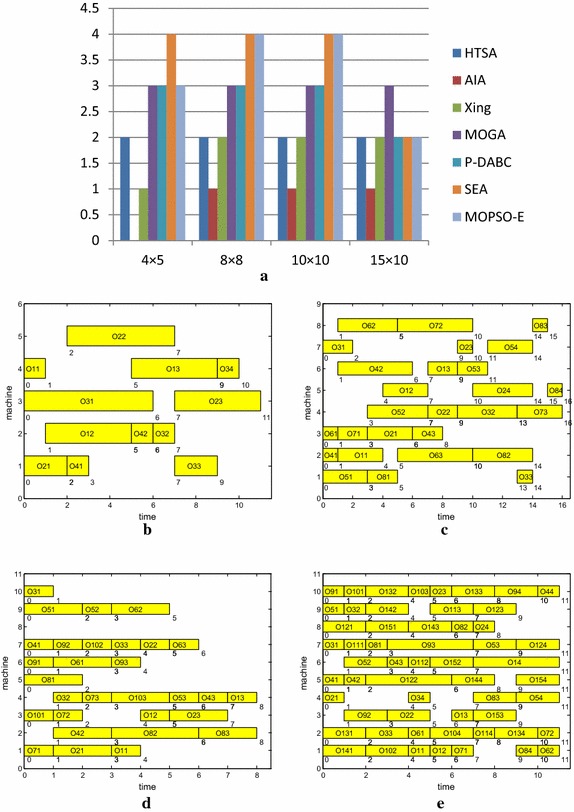
The Gantt chart of the solution of Kacem instances. **a** The number of non-dominated solutions for four Kacem instances, **b** 4 jobs 5 machines (*C*_*M*_ = 11, *W*_*T*_ = 32, *W*_*M*_ = 10) **c** 8 jobs 8 machines (*C*_*M*_ = 16, *W*_*T*_ = 73, *W*_*M*_ = 13), **d** 10 jobs 10 machines (*C*_*M*_ = 16, *W*_*T*_ = 73, *W*_*M*_ = 13), **e** 15 jobs 10 machines

Table 3 lists the number of the non-dominated solutions obtained by seven algorithms and Fig. 6a shows the comparison of the data in Table 3. From Table 3 and Fig. 6a, it is clear to see that the proposed algorithm obtains more non-dominated solutions than HTSA, AIA, Xing algorithm and P-DABC algorithm for all four instances. For the 4 jobs × 5 machines instance, SEA obtains one more non-dominated solutions than MOPSO algorithm but the paper lists no details of the non-dominated solutions, so it is lack of data for a further objective appraisal. For the 8 jobs × 8 machines instance, only P-DABC and MOPSO algorithm find four non-dominated solutions. For the 15 jobs × 10 machines instance, MOGA obtains three non-dominated solutions but two of them are dominated by the solutions of MOPSO algorithm. And only the HTSA, Xing algorithm and MOPSO algorithm find both non-dominated solutions (11, 91, 11) and (11 93 10). For all four Kacem instances, all non-dominated solutions are obtained by MOPSO algorithm and no one is dominated by the compared algorithm. Therefore, MOPSO algorithm has better comprehensive performance than the compared algorithms. The Gantt charts of four Kacem instances obtained by MOPSO algorithm are plotted in Fig. 6b–e.

**Table 3:** The number of non-dominated solutions for four Kacem instances

Kacem (m × n)	HTSA	AIA	Xing	MOGA	P-DABC	SEA	MOPSO
4 × 5	2	N/A	1	3	3	4	3
8 × 8	2	1	2	3	3	4	4
10 × 10	2	1	2	3	3	4	4
15 × 10	2	1	2	3	2	2	2

#### Test on the Brdata instances

The second category of 10 instances is from Brandimarte (Brdata instances) ranging from 10 jobs × 6 machines to 20 jobs × 15 machines and they are generated by a uniform distribution between given limits. Xing’s algorithm (Xing et al. 2009), MOGA (Wang et al. 2010), HTSA (Li et al. 2010), HSFLA (Li et al. 2012) and AIA (Bagheri et al. 2010) are used to compare with MOPSO algorithm. The data of compared algorithm are from the published paper (Li et al. 2010, 2012).

Table 4 lists the experimental results of non-dominated solutions with minimal makespan obtained by these algorithms. It is marked in italic type if the solution is dominated by other solutions. For MK02, MK05, MK06 and MK07 instances, the non-dominated solutions obtained by Xing’s algorithm are dominated by that obtained by MOPSO algorithm. For MK01 and MK03 instances, the non-dominated solutions obtained by MOGA are dominated by that obtained by MOPSO algorithm. For MK07 instances, the non-dominated solutions obtained by HTSA are dominated by that obtained by MOPSO algorithm. For MK02, MK05 and MK07 instances, the non-dominated solutions obtained by HSFLA are dominated by that obtained by MOPSO algorithm. For MK01, MK02, MK03, MK05, MK06, MK07, MK09, and MK10 instances, the non-dominated solutions obtained by AIA are dominated by that obtained by MOPSO algorithm. The only non-dominated solution of MK08 instance obtained by MOPSO algorithm is dominated by MOGA. From Table 4, we can clearly see that MOPSO algorithm obtains more high-quality solutions for ten Brdata instances.

**Table 4:** Results of the ten Brdata instances

Name	Xing	MOGA	HTSA	HSFLA	AIA	MOPSO
*MK01*						
*C*_M_	42	*40*	40	40	*40*	40
*W*_T_	162	*169*	167	165	*171*	167
*W*_M_	*42*	*36*	36	37	*36*	36
*MK02*						
*C*_M_	*28*	26	26	*26*	*26*	26
*W*_T_	*155*	151	151	*152*	*154*	151
*W*_M_	*28*	26	26	*26*	*26*	26
*MK03*						
*C*_M_	204	*204*	204	204	*204*	204
*W*_T_	852	*855*	852	852	*1207*	852
*W*_M_	204	*199*	204	204	*204*	204
*MK04*						
*C*_M_	68	66	61	62	60	61
*W*_T_	352	345	366	364	403	382
*W*_M_	67	63	61	61	60	60
*MK05*						
*C*_M_	*177*	173	172	*173*	*173*	173
*W*_T_	*702*	683	687	*685*	*686*	683
*W*_M_	*177*	173	172	*173*	*173*	173
*MK06*						
*C*_M_	*75*	62	65	64	*63*	62
*W*_T_	*431*	424	398	403	*470*	424
*W*_M_	*67*	55	62	55	*56*	55
*MK07*						
*C*_M_	150	139	*140*	*141*	*140*	139
*W*_T_	*717*	693	*695*	*696*	*695*	693
*W*_M_	*150*	139	*140*	*141*	*140*	139
*MK08*						
*C*_M_	523	523	523	523	523	*523*
*W*_T_	2524	2524	2524	2524	2524	*2524*
*W*_M_	523	515	523	523	523	*523*
*MK09*						
*C*_M_	311	311	310	311	*312*	310
*W*_T_	2374	2290	2294	2275	*2591*	2514
*W*_M_	299	299	301	299	*306*	299
*MK10*						
*C*_M_	227	214	214	215	*214*	214
*W*_T_	1989	2082	2053	1957	*2121*	2082
*W*_M_	221	204	210	198	*206*	204

Table 5 lists the best makespan (denoted as *C*_M_), the average computational time (denoted as Av(*CPU*)), the average best makespan (denoted as Av(*C*_M_)), the standard deviation (denoted as Std(*C*_M_)) obtained by MOPSO algorithm and some results obtained by SEA (Chiang and Lin 2013) and TSPCB (Li et al. 2011b). The improvements contrasted to SEA and TSPCB (respectively denoted as *imp*_1_ and *imp*_2_%) are calculated as follows:

$$imp\% = \frac{{C_{\text{M}}^{\text{com}} - C_{\text{M}}^{\text{pro}} }}{{C_{\text{M}}^{pro} }} \times 100\,{\text{\% }}$$

**Table 5:** Results of the ten Brdata instances

Name	SEA	TSPCB		MOPSO					
	*C*_M_	*C*_M_	Av(*CPU*)	*C*_M_	*Imp*_1_ %	*Imp*_2_ %	Av(*C*_M_)	Std(*C*_M_)	Av(*CPU*)
MK01	40	40	2.8	40	0	0	40.00	0	10.22
MK02	26	26	19.31	26	0	0	26.40	0.52	30.35
MK03	204	204	0.89	204	0	0	204.00	0	20.07
MK04	61	62	40.82	61	0	+1.61	62.35	0.47	58.22
MK05	173	172	20.23	173	0	−0.58	173.75	0.81	27.04
MK06	65	65	27.18	62	+4.62	+4.62	62.34	0.36	75.22
MK07	140	140	35.29	139	0	+0.71	139.30	0.46	52.51
MK08	523	523	4.65	523	0	0	523.75	0.68	45.20
MK09	311	310	70.38	310	+0.32	0	312.60	1.53	104.42
MK10	255	214	89.83	214	+16.08	0	214.55	0.65	156.73

where *C*_M_^com^ and *C*_M_^pro^ are the best makespan obtained by compared algorithm and obtained by our proposed algorithm respectively. *imp*% is the percentage of the improvement to the compared algorithm.

**Fig. 7:**
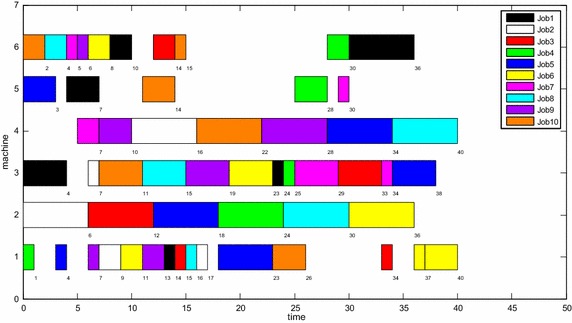
The Gantt chart of the solution of MK01 (*C*_*M*_ = 40, *W*_*T*_ = 167, *W*_*M*_ = 36)

**Fig. 8:**
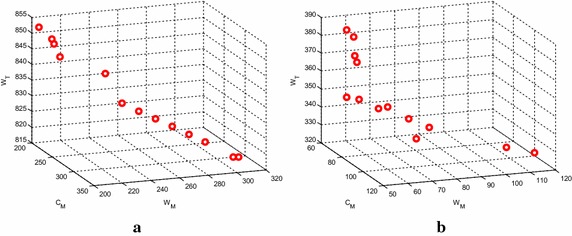
The approximate Pareto front of MK03 and MK04 obtained by MOPSO algorithm. **a** MK03, **b** MK04

From the data of *imp*_1_% in Table 5, we can see that *C*_M_ obtained by MOPSO algorithm has an improvement on MK06, MK09 and MK10 contrasted to that obtained by SEA, and no *C*_M_ is worse than that obtained by SEA. From the data of *imp*_2_%, MOPSO algorithm have better results on MK04, MK06 and MK07 than that obtained by TSPCB, and only for MK05, MOPSO algorithm obtains worse result than TSPCB. From Table 5, the values of Av(*C*_M_) for all Brdata instances are close to *C*_M_ and almost all the values of Std(*C*_M_)are less than 1. Therefore, we can conclude that MOPSO algorithm also has a stable searching ability. However, the MOPSO algorithm has more time-consuming than TSPCB. That maybe depends on different simulation environment and simulation language to some extent because TSPCB is implemented on Pentium IV 1.6 GHz processor in C++. The Gantt chart of one best solution obtained by the MOPSO algorithm for MK01 is shown in Fig. 7 and the approximate Pareto front of MK03 and MK04 obtained by MOPSO algorithm is shown in Fig. 8.

As PSO can memorize each particle’s experience and the population’s experience, PSO for multi-objective problem can track and memorize non-dominated solutions encountered by each particle (self experience) and the population (social experience), and a collaborative guiding way of self experience and social experience can balance exploration and exploitation and contribute to prevent premature. In our paper, the advantage of MOPSO is having many cognitive memories (no other algorithms have such memories) and a social memory to keep the diversity of non-dominated solutions and balance local search and global search. Then it is convenient for social memory to do a further research. This search mechanism can effectively avoid the premature and improve the solutions. It is verified by that the proposed algorithm obtains high-quality and more better solutions for most of Kacem instances and Brdata instances.

### Parameter sensitivity analysis

In this section, we should analyze the sensitivity of parameters. MK02 is applied to assess the performance of MOPSO with different parameter combinations. Four levels of the parameters *N*, *ω*, (*c*_1_, *c*_2_) and *c*_3_ are considered and experiments are designed by using the Taguchi method. The range of the parameters and the value of each factor level are presented in Table 6. The designed experiments of an orthogonal array are presented in Table 7.

**Table 6:** The factor level of parameters

Parameters	Value range	Factor level			
		1	2	3	4
*N*	50–200	50	100	150	200
*ω*	0.90–0.98	0.9	0.94	0.96	0.98
(*c*_1_, *c*_2_)	0.2–0.8	(0.2, 0.8)	(0.4, 0.6)	(0.6, 0.4)	(0.8, 0.2)
*c*_3_	0.001	0.001	0.01	0.05	0.1

**Table 7:** The orthogonal table of designed experiments

Experiment number	Factor level				Av(*C*_M_)
	*N*	*ω*	(*c*_1_, *c*_2_)	*c*_3_	
1	1	1	3	4	28.8
2	1	2	4	3	29.4
3	1	3	2	1	28.2
4	1	4	1	2	27.8
5	2	1	2	4	29.2
6	2	2	3	1	28.8
7	2	3	4	2	29.0
8	2	4	1	3	28.8
9	3	1	1	1	28.4
10	3	2	2	2	28.8
11	3	3	3	3	28.8
12	3	4	4	4	28.6
13	4	1	3	2	27.6
14	4	2	1	4	28.6
15	4	3	4	1	29.0
16	4	4	2	3	28.8

**Fig. 9:**
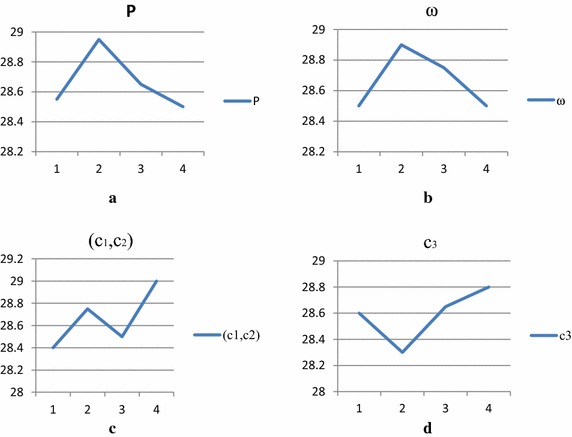
The trend of each factor level. **a** Trend of P. **b** Trend of w. **c** Trend of (c1, c2). **d** Trend of c3

Each designed experiment runs 5 times independently. The maximal iteration number is 100. Av(*C*_M_) denotes the average makespan of five runs. According to the results of Av(*C*_M_) in Table 7, the average Av(*C*_M_) of each factor level is presented in Table 8. In Table 8, ‘Delta’ denotes the maximal average Av(*C*_M_) minus the minimal average Av(*C*_M_) for each parameter and reflects the significance of each parameter. According to the Table 8, the trend of each factor level is illustrated in Fig. 9a–d and the effect on performance of each parameter is analyzed by Fig. 9. For comparisons of ‘Delta’, the parameters (*c*_1_, *c*_2_) rank first, and the parameter *c*_3_ ranks second. Therefore, the parameters (*c*_1_, *c*_2_) are the most significant factor on the performance of our algorithm.

**Table 8:** The Delta of each parameter

Factor	*N*	*ω*	(*c*_1_, *c*_2_)	*c*_3_
1	28.55	28.50	28.40	28.60
2	28.95	28.90	28.75	28.30
3	28.65	28.75	28.50	28.65
4	28.50	28.50	29.00	28.80
Delta	0.45	0.40	*0.60*	0.5

## Conclusions

In this paper, a multi-objective FJSP with three criteria is investigated to meet the requirements in manufacturing system and MOPSO algorithm is developed to address this problem. In MOPSO algorithm, a discrete version of PSO employing special discrete operators is proposed, and personal-best archives and global-best archive, which is updated by non-dominated archive update strategy and is respectively used to select personal-best positions and global-best position, are developed to preserve non-dominated positions. Cognitive memories and social memory can keep the diversity of non-dominated solutions and balance local search and global search. Additionally, variable neighborhood search integrating three neighborhoods on the global-best archive is applied to improve the exploiting capability. MOPSO algorithm is evaluated on Kacem instances and Brdata instances, and compared with some published algorithms. Computational experiments demonstrate that the MOPSO algorithm have a better comprehensive performance than other algorithms to solve multi-objective FJSP.

Research work should be continued in the future and it includes the followings: Firstly, the theory of multi-objective optimization should be developed to support the study of multi-objective FJSP. Secondly, the FJSP model also should be developed or rebuilt to meet the dynamic and varied environment and requirements. Finally, more efficient algorithm or strategy should be studied for solving the difficult FJSP problem.
